# Challenges posed by climate hazards to cardiovascular health and cardiac intensive care: implications for mitigation and adaptation

**DOI:** 10.1093/ehjacc/zuae113

**Published:** 2024-10-28

**Authors:** Thomas Münzel, Haitham Khraishah, Alexandra Schneider, Jos Lelieveld, Andreas Daiber, Sanjay Rajagopalan

**Affiliations:** University Medical Center Mainz, Department of Cardiology, Johannes Gutenberg University, Langenbeckstr. 1, 55131 Mainz, Germany; Harrington Heart and Vascular Institute, University Hospitals at Case Western Reserve University, Cleveland, OH, USA; Institute of Epidemiology, Helmholtz Zentrum München – German Research Center for Environmental Health, Neuherberg, Germany; Max Planck Institute for Chemistry, 55128 Mainz, Germany; University Medical Center Mainz, Department of Cardiology, Johannes Gutenberg University, Langenbeckstr. 1, 55131 Mainz, Germany; Case Cardiovascular Research Institute, Case Western Reserve University School of Medicine and University Hospitals Harrington Heart and Vascular Institute, 11100 Euclid Ave, Cleveland, OH 44106, USA

**Keywords:** Climate change, Heatwave, Non-optimal temperature, Cardiovascular disease

## Abstract

Global warming, driven by increased greenhouse gas emissions, has led to unprecedented extreme weather events, contributing to higher morbidity and mortality rates from a variety of health conditions, including cardiovascular disease (CVD). The disruption of multiple planetary boundaries has increased the probability of connected, cascading, and catastrophic disasters with magnified health impacts on vulnerable populations. While the impact of climate change can be manifold, non-optimal air temperatures (NOTs) pose significant health risks from cardiovascular events. Vulnerable populations, especially those with pre-existing CVD, face increased risks of acute cardiovascular events during NOT. Factors such as age, socio-economic status, minority populations, and environmental conditions (especially air pollution) amplify these risks. With rising global surface temperatures, the frequency and intensity of heatwaves and cold spells are expected to increase, emphasizing the need to address their health impacts. The World Health Organization recommends implementing heat–health action plans, which include early warning systems, public education on recognizing heat-related symptoms, and guidelines for adjusting medications during heatwaves. Additionally, intensive care units must be prepared to handle increased patient loads and the specific challenges posed by extreme heat. Comprehensive and proactive adaptation and mitigation strategies with health as a primary consideration and measures to enhance resilience are essential to protect vulnerable populations and reduce the health burden associated with NOTs. The current educational review will explore the impact on cardiovascular events, future health projections, pathophysiology, drug interactions, and intensive care challenges and recommend actions for effective patient care.

## Introduction

The Earth’s average surface temperature has increased by about 1.2°C (2.2°F) since the late 19th century with most of this warming occurring in the last few decades, resulting in the 24 of the hottest years since 2000.^1^ This period also has experienced an unprecedented frequency, intensity, and duration of extreme temperature events and intensity of natural disasters, such as storms, forest fires, and floods. From 1998 to 2017, these climate-related and geophysical disasters resulted in 1.3 million deaths and affected 4.4 billion people.^2^ These trends are expected to worsen in the coming years, given the continual global warming and the greater vulnerability of patients with multiple risk factors for cardiovascular disease (CVD).^3^ According to the World Health Organization (WHO), ∼3.6 billion people live in climate-vulnerable areas. Individuals in climate-vulnerable areas may be exposed to a multitude of climate risks, especially among the elderly, including rises in diarrhoeal diseases, malaria, dengue, coastal flooding, and childhood stunting. Climate risks are particularly high for low-income and marginalized populations, especially in countries that have contributed the least to carbon emissions. Non-optimal temperatures (NOTs) present significant health risks, whether during isolated hot days, prolonged heatwaves, or severe cold spells. According to the Global Burden of Diseases, Injuries, and Risk Factors Study (GBD), in 2021, 1.17 million [95% confidence interval (CI): 1.07–1.29 million] cardiovascular deaths and 1.81 million (95% CI: 1.65–1.97 million) deaths overall were estimated as attributable to NOTs.^4^ The all-cause disability-adjusted life years due to NOT were 420 per 100 000 (95% CI: 383–461 per 100 000). Heat-related excess deaths were primarily in Europe, while sub-Saharan Africa had the highest estimates of cold-related excess deaths. Individuals with pre-existing health issues, particularly those suffering from pre-existing health conditions, including CVD, are especially vulnerable, resulting in increased emergency room visits and hospital admissions.^2^ Moreover, adverse environmental conditions, particularly air pollution, can compound the health impacts of high temperatures, leading to more severe outcomes.^3,5^ Population growth, ageing, urbanization, and evolving socio-economic development pathways amplify the vulnerability to NOTs including heat stress.^6^

Thus, effective public health strategies and policies are essential to mitigate these risks, thereby protecting vulnerable populations. This educational review will present current evidence of the adverse effects of NOT on CVD events and factors contributing to temperature vulnerability. It will explore potential pathophysiological mechanisms underlying the cardiovascular effects of heat, as well as the interaction between heat and cardiovascular drugs. The impact of specific challenges in cardiac intensive care due to heatwaves will be highlighted, and recommendations on how medical physicians can engage in heat–health action plans (HHAPs), thereby delivering effective patient care during heat exposure. Adaptation and mitigation to climate change with a focus on the healthcare industry will also be discussed.

## Planetary boundaries, tipping points, and climate disasters

**FIGURE 1:**
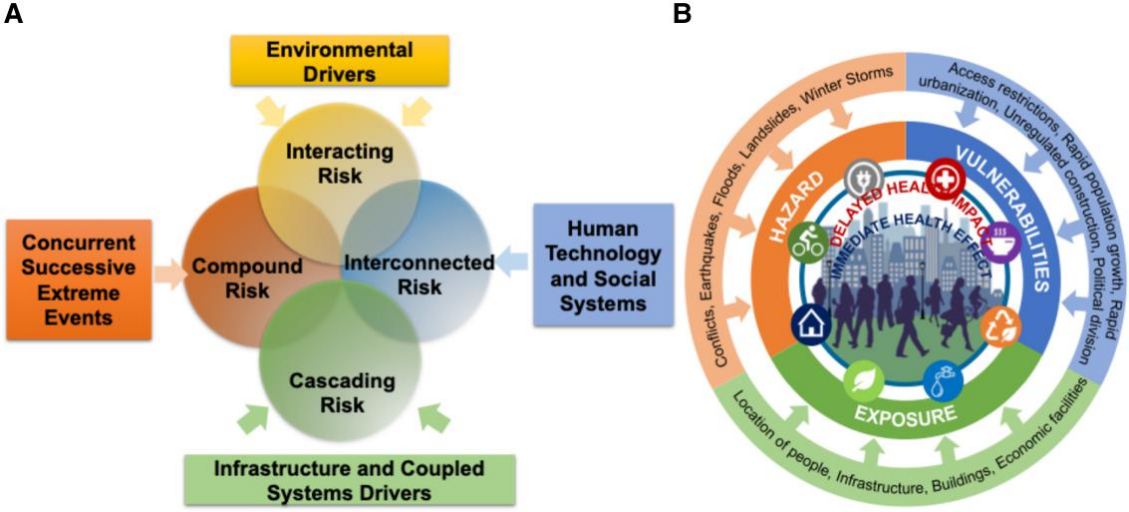
(*A*) Impact of climate hazards on coupled interconnected human environment and technological systems resulting in magnified impact. (*B*) The impact of climate hazards and exposures on health that are magnified, when connected provisioning systems such as food, water, hospitals, transportation, housing, waste and other infrastructure is disrupted Both immediate and delayed health effects are experienced. The graph on the right is adapted from Understanding Compound, Interconnected, Interacting, and Cascading Risks: A Holistic Framework. Risk Analysis, Vol. 38, No. 11, 2018.

The planetary boundaries framework identifies nine critical processes essential for maintaining the Earth’s ecosystem stability and safe human existence.^7,8^ These include climate change, biosphere integrity, land-system change, freshwater use, biogeochemical flows, ocean acidification, atmospheric aerosol loading, stratospheric ozone depletion, and introduction of emerging exposures.^9^ Alarmingly, an analysis reported in 2023 found that six of these nine boundaries have been transgressed due to human activities, indicating that the planet has moved outside the safe operating zone for humanity in those areas.^9^ Violating these boundaries increases the risk of large-scale abrupt or irreversible environmental changes can have severe consequences for human health and well-being.^9^ More recently, the earth systems boundary concept has been promulgated and includes the ‘3I’ framework or interspecies justice, intergenerational justice, and intragenerational justice.^10^ The current carbon dioxide (CO_2_) level of 420 p.p.b. already exceeds the planetary boundary limit of 350 p.p.m. CO_2_. At current emission rates, the budget for 1.5°C could be exhausted within the next decade, and the 2°C budget not long after. A 2°C rise in temperature would have serious global consequences, with a very high likelihood of multiple tipping points, precipitating interconnected cascading disasters with severe health impacts. In order to understand systemic risk due to climate hazards and tipping points in planetary spheres, including the ecosphere, hydrosphere, geosphere, atmosphere and cryosphere, it is relevant to explore the nature of complex adaptive systems, including properties of systems, including interconnectedness, path dependency, generativity, dynamism, and feedback loops. The non-linear nature of system interactions in highly complex adaptive systems often results in unintended and unpredictable consequences, often widely separated in space and time, complicating interpretations of cause and effect.^11,12^ Many complex systems thus carry inherent risks to perturbations beyond a certain tipping point when disasters occur. The risks seen in complex system are often referred to differently, in disparate fields of study and are worthwhile enunciating. The term cascading risk is typically used to describe risks in anthropogenic domains, such as infrastructural risks in tightly coupled organizational components. Cascading risk can, therefore, only occur in interconnected human, environmental, and technological systems, resulting in a magnified impact. Compound risk, on the other hand, is typically in the environmental context and to the concurrence of concurrent or successive extreme events. Interacting risks focus on the aspect of how climate hazards may interact with vulnerability to create disaster risk. *Figure 1* presents a socio-ecological-infrastructural systems framework, with key provisioning systems of food, energy, transportation, housing, water, waste management, and greenery/green infrastructure.^13,14^ While essential for human living, provisioning systems counterintuitively generate multi-scale risks related to food scarcity, power outages, destruction of housing, lack of water and paralysis of transportation during a climate-related disaster. The term vulnerability, as used in this paper in physical, infrastructural, social, and economic domains, often segregated in space, may increase the susceptibility of an individual, a community, or systems to determine the impacts of hazards.

## Climate vulnerability, hazards, and exposures

**FIGURE 2:**
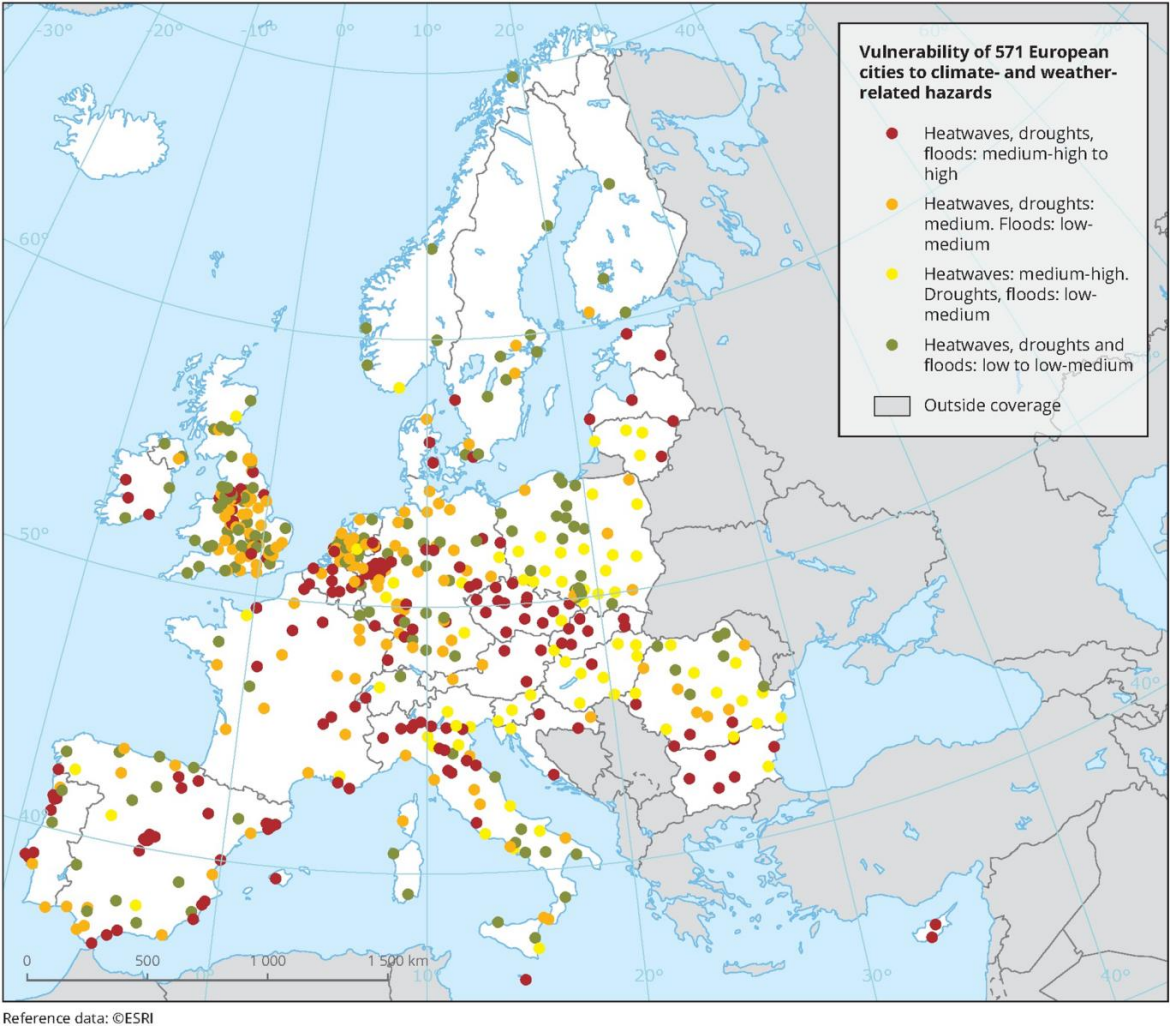
Within the framework of the Horizon2020 research project RAMSES, the vulnerability of 571 European cities was assessed according to several indicators from various domains, including human capital, governance and institutions, socio-economic conditions, build environment, and natural capital. The cities were clustered according to their vulnerability to heatwaves, flooding, and droughts.

The IPCC Fifth Assessment Report of 2014 (AR5) defines vulnerability as one of the 3 core components determining climate risks, the other two being hazard and exposure (IPCC, 2014). In AR5, vulnerability is defined as ‘the propensity or predisposition to be adversely affected’, encompassing ‘a variety of concepts and elements including sensitivity or susceptibility to harm, and lack of capacity to cope and adapt’ (IPCC, 2014, Glossary). Therefore, albeit some earlier studies consider vulnerability as a function of sensitivity, adaptive capacity, and exposure. Indicator-based Vulnerability Assessments, or IBVA, have usually attempted to define the processes affecting vulnerability within specific domains of the system under analysis. These aspects are often individually assessed and combined into vulnerability ‘indices’ or ‘scores’.^15,16^ At the urban level, IBVAs typically focus on: (i) cities or sub-city domains such as counties or census tracts that are then evaluated for their vulnerability, and (ii) alternately, the impact on infrastructure provisioning systems, including specific communities and social groups can be evaluated. In the USA, the Climate and Economic Justice Screening Tool (CEJST) has an interactive map and uses data set indicators of burdens in eight categories: climate change, energy, health, housing, legacy pollution, transportation, water and wastewater, and workforce development.^17^ The tool uses this information to identify communities that are experiencing these burdens. Climate-vulnerable areas (census tracts) are communities that are identified as disadvantaged if they are in census tracts that are at or above the 90th percentile for expected agriculture loss rate OR expected building loss rate OR expected population loss rate OR projected flood risk OR projected wildfire risk AND are at or above the 65th percentile for low income.^18^*Figure 2* depicts the vulnerability of 571 European cities, according to several indicators, including human capital, governance and institutional, socio-economic, built environment, and natural capital. The cities were the rated according to their vulnerability to heatwaves, flooding, and droughts.^16^

## The impact of non-optimal temperature and climate hazards on cardiovascular outcomes

The Lancet Countdown on health and climate change summarized the effects of extreme temperatures, among many other consequences of climate change, on health and disease, including CVD.^19^ A 2021 global analysis estimated that >5 million deaths are associated with NOTs annually.^3^ A substantial proportion of this is related to cardiovascular events. Meta-analyses also confirm that cardiovascular illness is the leading cause of death during heatwaves.^4,14^ In a recent systematic review, out of 492 observational studies that met the criteria almost 392 focused on NOT and ozone pollution (182 NOT and 210 ground-level ozone), while the remainder focused on wildfire smoke and extreme weather events, such as hurricanes, dust storms, and droughts.^20^ The studies were almost all from high-income countries (HICs) and middle-income countries. The strength of evidence was rated as sufficient for extreme temperatures, ozone, tropical storms, hurricanes, and cyclones and dust storms. Exposure to NOT was associated with increased cardiovascular mortality and morbidity, but the magnitude varied with temperature and duration of exposure. Ground-level ozone amplified the risk with higher temperatures and *vice versa*.^20^ Extreme weather events, such as hurricanes, were associated with increased cardiovascular risk that persisted for many months after the initial event. Evidence was rated as limited for wildfire smoke and inadequate for drought and mudslides.^20^

Climate change has also increased the frequency of dust storms and wildfires in addition to heatwaves, and these events often occur together.^21–26^ Dust storms consist of large (PM_10_), coarse (PM_2.5–10_), and fine (PM_2.5_) particulate matter. A systematic review and meta-analysis showed a 0.27% increase in all-cause mortality when comparing dust days with non-dust days.^27^ A meta-analysis of studies of dust exposure in Asia showed a 2.33% relative increase in combined respiratory and circulatory deaths when comparing dust days with non-dust days.^28^ In Taipei, Taiwan, researchers found a relative increase of 26% in overall emergency admissions for CVDs during dust-event days.^29^ Specifically, there was a 35 and 20% increase in the relative risk (RR) of ischaemic heart disease (IHD) and stroke emergency visits, respectively, during dust periods compared with pre-dusk periods. Several other studies have shown an association between exposure to dust and the risk of stroke (both ischaemic and haemorrhagic) and heart failure (HF) hospitalizations.^2^

Both low and high temperatures contribute to cardiovascular morbidity and mortality.^30,31^ Although in GBD, the greatest mortality burden is associated with low rather than high temperatures.^32^ Globally, ∼5 million deaths were attributable to NOT per year, accounting for 9.43% of all deaths; 8.52% were cold related, and 0.91% were heat related. Thus, the ratio of cold-related deaths vs. heat-related deaths was around 9.36. Due to the increase in heatwaves, a net reduction in the overall ratio has been observed.^9^ With the warming of 1.5°C, 2°C, and 3°C of global warming, heat-related mortality in 800 locations across 50 countries/areas increased by 0.5, 1.0, and 2.5%, respectively.^33^ Despite a projected decrease in cold-related mortality due to progressive warming alone, population ageing will mostly counteract this trend, leading to a net increase in cold-related mortality by 0.1–0.4% at 1.5–3°C global warming.^33^ Most studies focus on high temperatures focus on the independent effect of a daily high temperature, with the added effect due to the duration of sustained heat for several consecutive days or heatwaves on cardiovascular mortality being relatively less common. In this regard, the definition of heatwaves, which also include duration, may markedly influence its impact. A study in China found that heatwaves ≥5 days had the greatest risk, with an increase of 18% (95% CI: 6–31%) in the overall population, 24% (95% CI: 10–39%) in an older group ≥65 years.^34^

As observed in many previous studies, a population exposed to warmer climates will reduce the cold-related mortality burden that could outnumber the expected increase in heat-related mortality burden.

## How does non-optimal temperature contribute to cardiovascular events?

**FIGURE 3:**
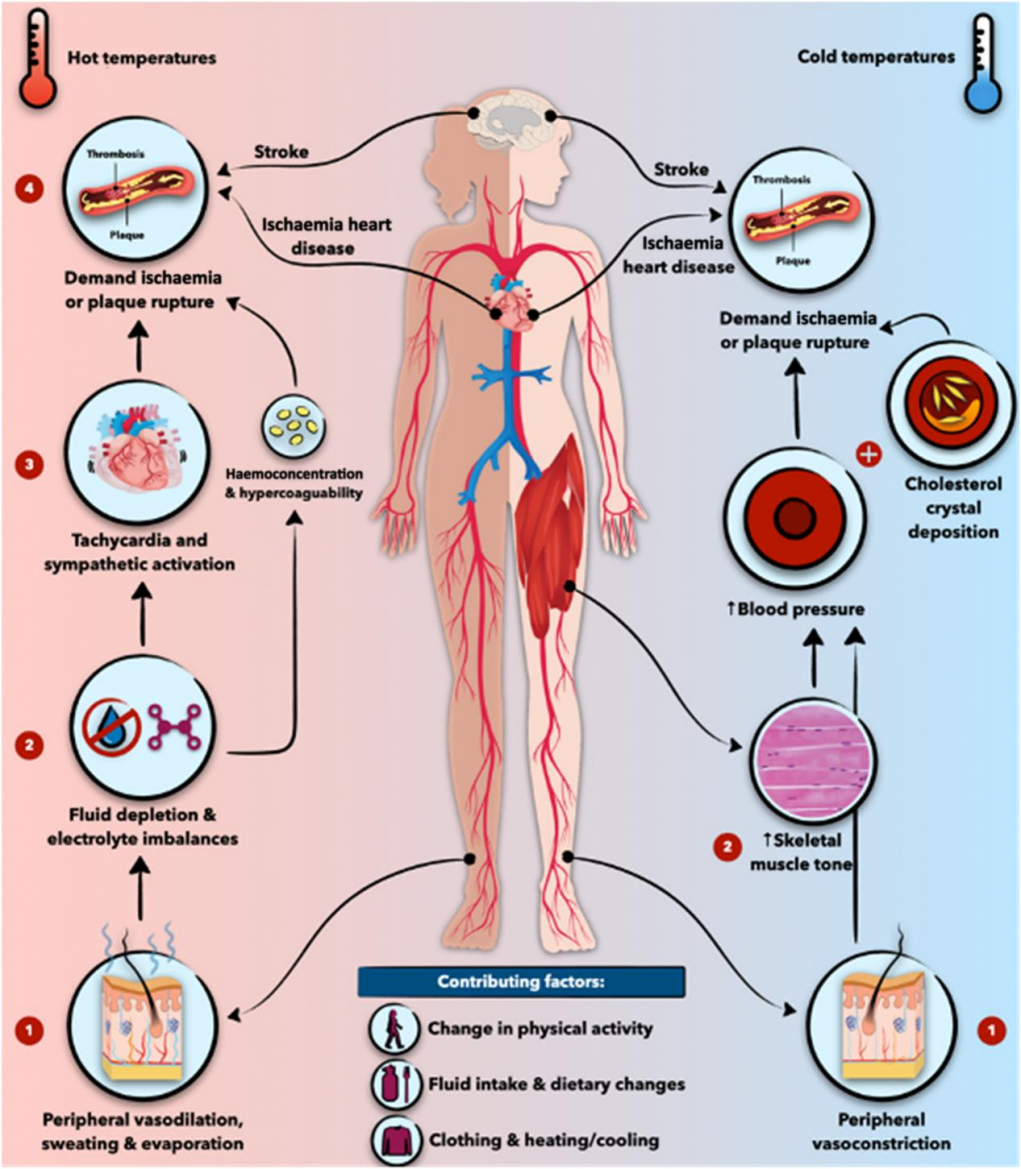
Pathophysiological pathways involved in mediating the effects of non-optimal temperature on cardiovascular disease. To lower core body temperature, heat exposure induces peripheral vasodilatation and sweating. These responses can result in dehydration, haemoconcentration, and electrolyte imbalance, and can trigger sympathetic activation and tachycardia. In individuals with pre-existing cardiovascular conditions, these sequences of events may lead to demand ischaemia or atherosclerotic plaque rupture, potentially causing myocardial infarction and/or stroke. Conversely, exposure to cold triggers sympathetic activation, peripheral vasoconstriction, and increased muscle tone, raising blood pressure levels. Cold exposure can also encourage cholesterol crystal deposition within atherosclerotic plaques. The combined effects of sympathetic activation, elevated blood pressure, and cholesterol crystallization can result in demand ischaemia and/or atherosclerotic plaque rupture. The figure was adopted from an in-press article “Climate Change and Cardiovascular Disease: Who is Vulnerable?” by Khraishah et al published in Arteriosclerosis, Thrombosis, and Vascular Biology (ATVB).

The human body responds to heat stress primarily by redistributing blood flow towards the skin (vasodilation) to facilitate heat transfer from muscles and secreting sweat onto the skin, which evaporates and removes body heat.^35^ The brain regulates these heat loss responses, with additional input from temperature-sensitive nerve cells in the skin and body.^36^ This regulation can also be influenced by non-thermal signals such as dehydration, metaboreceptors (responding to metabolic products from exercising muscles), and cytokines.^36^ These homeostatic responses are crucial to limiting core temperature increases and can vary among individuals, especially those with pre-existing medical conditions, potentially causing adverse effects. Cutaneous vasodilation increases cardiac demand while decreasing the filling pressure of the heart.^37^ This forces the heart to pump harder and faster, raising oxygen demand in coronary tissue. For those with heart conditions, this can lead to a mismatch between high cardiac oxygen demand and compromised delivery. A sustained mismatch can result in cardiac ischaemia, infarction, and cardiovascular collapse.^38^*Figure 3* outlines the principal mechanisms involved in NOT-mediated cardiovascular events. Cardiovascular strain from heat stress is a significant concern, especially during heat extremes, with older adults more likely to die from cardiovascular events than any other heat-related cause.^6,39^ Additionally, sweating can lead to dehydration, decreasing blood volume, and exacerbating cardiovascular strain.^40^ Severe dehydration can cause acute kidney injury and failure, while chronic dehydration can lead to kidney fibrosis and chronic kidney disease, particularly among outdoor workers.^41^ This further worsens CVD and is frequently reported during or after hot weather events.^41^ The body’s thermoregulatory capacity can be overwhelmed by extreme heat stress, leading to overheating and potentially fatal heatstroke.^42^ High internal temperatures (39–40°C), combined with ischaemia and oxidative stress from blood redistribution, can damage cells, tissues, and organs, with the brain, heart, kidneys, intestines, liver, and lungs at greatest risk.^41^ Exertional heatstroke causes lung damage, including pulmonary oedema and acute respiratory distress syndrome, especially in those with pre-existing respiratory conditions.^43^ This, along with hyperventilation and increased air pollution during heatwaves, is responsible for significant heatwave-related mortality and morbidity, second only to CVD.^4,6^ Heat-derived injuries remain hazardous, even after cooling the body, with long-term cognitive and organ dysfunction. Most heat-related hospital admissions occur within 24 h of heat events, but repercussions can last for years, significantly increasing the risk of death for decades post-injury.^44^

Exposure to cold triggers the activation of sympathetic tone, leading to the constriction of both skin and skeletal muscle vessels. This response helps conserve and may even generate heat. However, it also results in an elevation in blood pressure, thereby increasing afterload and cardiac oxygen demand—both of which can exacerbate HF. Like heat, cold periods induce a state of hypercoagulability due to increased viscosity, haemoconcentration, and clotting abnormalities.^24^

## Non-optimal temperature and cardiovascular events

### Ischaemic heart disease

**FIGURE 4:**
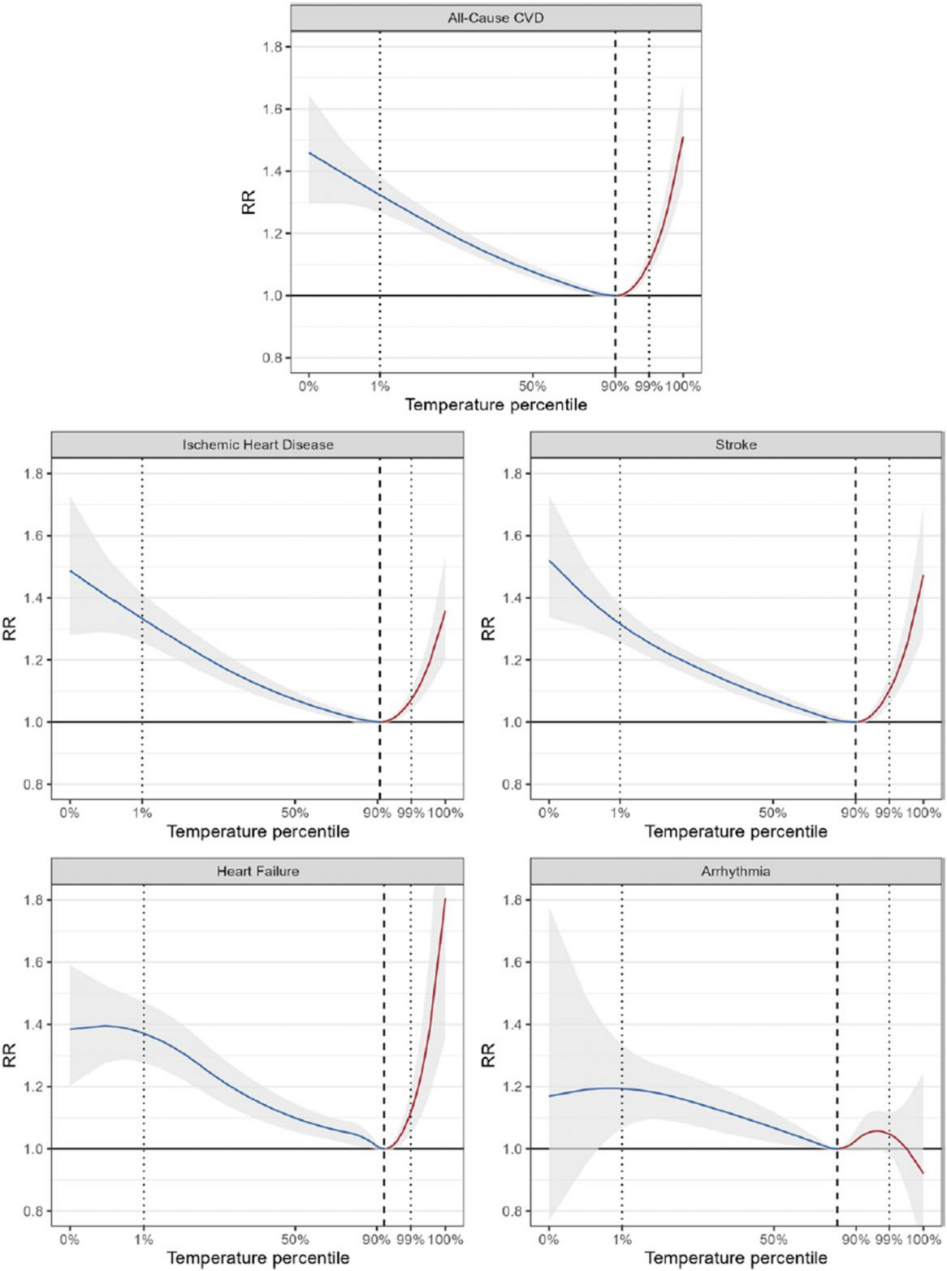
Pooled exposure–response relationships. Relationships are described as temperature percentiles and relative risk of different cardiovascular causes of death: all-cause cardiovascular (567 cities), ischaemic heart disease (567 cities), stroke (567 cities), heart failure (524 cities), and arrhythmia (441 cities). Dashed line indicates the minimum mortality temperature. Dotted line indicates the 1st percentile (extreme cold) and the 99th percentile (extreme heat). *X*-axis was transformed from absolute temperatures (°C) to percentiles to enable a comparative application of the association. Cardiovascular disease indicates cardiovascular disease (with permission from Alahmad *et al*.^45^).

Ischaemic heart disease remains the leading cause of cardiovascular mortality. While the impact of low temperatures on myocardial infarction (MI) hospitalizations is well-documented, the effects of heat on acute MI are now increasingly becoming apparent. In a very large multi-country, multi-city collaborative (MCC) network involving 567 cities from 27 countries, the relationship between temperatures and IHD events was studied. A total of 32 154 935 all-cause CVD deaths, 11 745 880 IHD deaths, 9 351 312 stroke deaths, 3 673 723 HF deaths, and 670 859 arrhythmia deaths, covering largely overlapping years between 1979 and 2019 were evaluated. Overall, a non-linear relationship between IHD outcomes, including arrhythmias, and temperature was noted in both hot and cold temperature ranges (*Figure 4*).^45^ The risk of death increased gradually for the cold temperatures below the minimum mortality temperature (MMT). In contrast, the slope for hot temperatures was slightly steeper, especially with HF where the RRs appear to escalate quickly. The pooled RRs of death associated with extreme heat (99th percentile vs. MMT) from IHD, stroke, and HF were 1.07 (95% CI: 1.04–1.10), 1.10 (95% CI: 1.06–1.15), and 1.12 (95% CI: 1.05–1.19), respectively (*Figure 4*). Meanwhile, the pooled RRs of death associated with extreme cold (1st percentile vs. MMT) from IHD, stroke, and HF were 1.33 (95% CI: 1.26–1.41), 1.32 (95% CI: 1.26–1.38), and 1.37 (95% CI: 1.28–1.47), respectively. The risk of dying from arrhythmias was associated with greater uncertainty and a smaller effect size estimates for extreme heat. Several limitations of this large study should be acknowledged, including under-representations of large climate-vulnerable regions of the world including South Asia, the Middle East, and Africa. Additionally, there are substantial differences in baseline rates of CVD, access to healthcare and urbanization characteristics that may additionally influence outcomes in these areas.

The relative importance of heat-related MI hospitalizations is expected to become more apparent with the current trajectory of climate change. A previous study compared the incidence of temperature-related MI during 1987–2000 and 2001–14.^46^ Myocardial infarction was triggered solely by cold exposure during the earlier period, but the RR of heat-related MI significantly increased in the latter period. This increased susceptibility to heat was more pronounced in patients with diabetes and, hyperlipidaemia, highlighting the importance of identifying NOTs as a risk factor for CVD, especially in patients with pre-existing risk factors for CVD. Smaller studies have also reported significant increases in coronary artery disease–related hospitalizations and emergency department visits during heatwaves.^47^ A meta-analysis previously demonstrated a 2.8% increased risk of coronary heart disease for every 1°C rise above reference temperature.^48^ Prior studies have suggested that the health impacts of exposure to extreme low temperatures usually persist longer (up to 2 weeks or more) than those from extreme heat events, which typically last for 2–3 days.^49,50^ As extreme heat events become more frequent due to global warming, the impact of heat on IHD is expected to grow.

### Stroke

Prior analyses have indicated a significant link between extreme temperatures and stroke risk. A meta-analysis of over 2 million stroke events found that a 1°C increase in temperature raised stroke risk by 1.13%, while a 1°C decrease raised it by 1.2%.^51^ This risk is higher for those aged 65 and older. A prior meta-analysis of 21 studies with 476 511 patients found no significant link between hot temperatures and ischaemic stroke.^52^ Conversely, a study in Seoul found a positive association between mean temperature and ischaemic stroke.^53^ A more recent patient level analysis from the multi-country multi-city study that included a total of 3.4 million ischaemic strokes and 2.5 million haemorrhagic stroke deaths from 522 cities in 25 countries, demonstrated an impact of extreme temperatures on both ischaemic and haemorrhagic strokes.^54^ Extreme hot and cold days (defined as 2.5% hottest and coldest days), contributed 9.1 and 2.2 excess deaths, respectively, for every 1000 ischaemic strokes, and 11.2 and 0.7 excess deaths, respectively, for haemorrhagic strokes. Countries with low gross domestic product (GDP) per capita were at higher risk of heat-related haemorrhagic stroke mortality than countries with high GDP per capita. However, the same limitations of the MCC analysis in not including large climate-vulnerable areas of the world stand. In addition, recent results indicate a significant increase in stroke risk on days with extreme night-time heat [97.5% percentile of hot night excess (HNE); odds ratio 1.07, 95% CI: 1.01–1.15] during the full study period. When comparing the results for 2013–20 with the results for 2006–12, there was a significant increase in HNE-related risk for all strokes and specifically for ischaemic strokes during the more recent period. Furthermore, older individuals, females, and patients with mild stroke symptoms exhibited a significantly increased vulnerability to night-time heat.^55^ A study in China found increased heat-related mortality risks for total stroke (1.54), ischaemic stroke (1.63), and haemorrhagic stroke (1.36).^56^

### Heart failure

Research on the relationship between extreme temperatures and HF is limited with no studies according to the type of HF. Previous studies have shown increased HF admissions and mortality during winter months.^57,58^ A large study using multi-country data found that extreme heat led to 2.6 excess deaths per 1000 HF deaths, with a 12% increase in HF-related mortality.^45^ A large study from Japan reported that cold temperatures significantly increased the risk of HF hospitalization (RR = 1.571, 95% CI: 1.487–1.660) compared with IHD (RR = 1.119, 95% CI: 1.040–1.204) or stroke (RR = 1.107, 95% CI: 1.062–1.155). Extreme heat also increased the risk of HF but at an RR of 1.030 (95% CI: 1.007–1.054). Sub-group analysis showed that the age group ≥85 years was more vulnerable to these NOT risks.^59^

### Arrhythmia

The relationship between extreme heat and arrhythmia-related mortality or admissions is inconsistent, likely due to using arrhythmia as an umbrella term instead of looking at individual diagnoses. A South Korean study found that each 1°C increase in the diurnal temperature range was associated with a 1.84% increase in cardiac arrhythmias. Another study showed that a 1°C increase in same-day temperature raised the odds of ventricular ectopy episodes by 1.10.^60^ However, a study of 345 052 arrhythmia admissions in Ontario found no association between extreme temperatures and arrhythmia-related outcomes.^61^ Similarly, a London study found no significant increase in the risk of implanted cardiac defibrillator activation during higher temperatures.^62^ Detailed analysis of the relationship between NOT and ventricular vs. atrial arrhythmias requires further investigation.

## Air pollution and non-optimal temperature interactions on cardiac health

Air pollutants have been recognized since antiquity, but their sources and composition have evolved with industrialization and urbanization, leading to significant public health concerns.^63^ Climate is a major determinant of air quality and vice versa, anthropogenic air pollution is a major contributor to climate change.^64^ The effects of air pollution are attributable to its complex chemistry and size, which are in turn, determined by their sources and prevalent atmospheric conditions.^65,66^ The latter is profoundly modified by weather conditions and climate, necessitating a re-exploration of some of the previously held precepts about this ancient risk factor. The sources and chemistry of air pollution have been previously discussed extensively and will not be reviewed here but pollutants in both the particulate and gaseous phase characterize air pollution.^67–72^ Recent studies established that PM_2.5_ plus ozone is responsible for excess mortality of 8.4 million people/year.^40,73^ In addition, exposure to PM_2.5_ reduces the global life expectancy by 2.9 years.^74^

**FIGURE 5:**
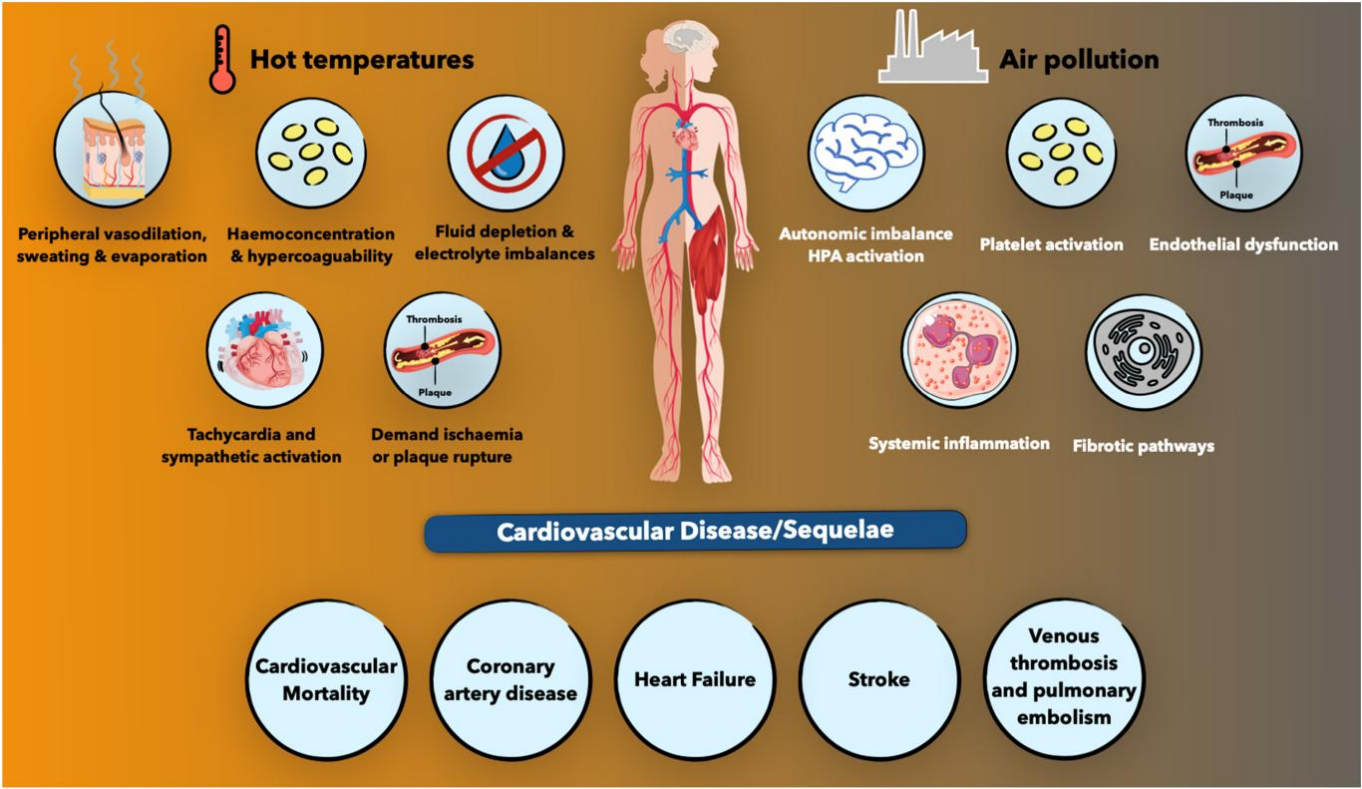
Pathophysiological and clinical consequences of simultaneous exposure to air pollution and heat.

Increased heat facilitates the photochemical formation and transformation of air pollutants, hindering their dilution and dispersal.^2^ Rising temperatures also increase the oxidizing potential of the atmospheric components, to produce more sulphate particles.^75–77^ Conversely, temperature inversions can also result in episodes of extreme air pollution, which may exacerbate cardiovascular toxicity of air pollution. The increased warming of the planet has resulted in marked changes in precipitation patterns, resulting in drier conditions in many parts of the world that can increase the frequency and intensity of wildfires and dust storms. The concept of ‘climate penalty’ refers to the enhanced formation of secondary pollutants such as ground-level ozone (tropospheric) with rising temperatures and altered meteorologic conditions caused by climate change. Several studies have now demonstrated an increase in cardiovascular mortality with both short-term elevations in ground level ozone and long-term exposure to ozone with an interaction between elevated levels of ozone and cardiovascular mortality in some of these studies.^78–80^ A time-series study in 2017, examined the two-way effect modifications of higher air temperature and pollution in eight European cities. A greater effect of pollution measures on total and cardiovascular mortality was noted on days with higher air temperature.^81^ Similarly, ozone-related mortality was found to be higher on warmer days. While the precise mechanisms for this interaction are unclear, from a cardiovascular perspective, this may relate to a synergistic effect of air pollutants and higher air temperature on the cardiovascular system, resulting in systemic oxidative stress, inflammation, endothelial dysfunction, and increased thrombosis. A previous systematic review demonstrated a robust interactive effect between exposure to heat and air pollutants, with the strongest evidence for exposure to ozone and PM_2.5_. The authors found a moderate quality of evidence of a dose–response relationship between heat and air pollution, with good evidence for a combined effect on all-cause and cardiovascular mortality.^82^ A study in California estimated the effect of co-exposure to extreme heat and fine particulate matter and found the combined effects of both, were more than the sum of individual effects of extreme temperature and PM_2.5_.^83^ All-cause mortality risk increased by 6.1% (95% CI: 4.1–8.1) on extreme maximum temperature-only days and 5.0% (95% CI: 3.0–8.0) on extreme PM_2.5_-only days, compared with a risk of 21.0% (95% CI: 6.6–37.3) on days with both extreme maximum temperature and PM_2.5_. Increased risk of cardiovascular and respiratory mortality was 29.9% (95% CI: 3.3–63.3) and 38.0% (95% CI: −12.5 to 117.7), respectively. In a large time-stratified case-crossover study of 202 678 MI deaths in Jiangsu province, China, the odds ratio of MI mortality associated with heatwaves and cold spells ranged from 1.18 (95% CI: 1.14–1.21) to 1.74 (1.66–1.83), and 1.04 (1.02–1.06) to 1.12 (1.07–1.18), respectively.^76^ A 1-day lag in exposure to PM_2.5_ was associated with increased odds of MI mortality, which attenuated at higher exposures. The study estimated that up to 2.8% of the MI deaths were attributable to exposure to extreme temperature events and PM_2.5_ at levels exceeding the interim target 3 value (37.5 μg/m^3^) of WHO air quality guidelines. Rai *et al*.^75^ investigated the relationship between high temperatures and CVD modified by PM_10_, O_3_, and NO_2_. All three pollutants increased the risk of heat-related CVD mortality, with the effect being most pronounced for O_3_ and NO_2_. Overall, higher levels of air pollution were observed to amplify the detrimental effects of high temperatures on CVD mortality. A white paper from the European Union concluded that there was high potential for co-benefit on health and prevention of diseases in considering air pollution in heat health prevention and protection. Even if the RRs of air temperature and air pollution may be small compared with other individual or lifestyle risk factors, the population impact is very large.^84^*Figure 5* details the distinct pathways involved in air pollution and NOT-mediated cardiovascular events.

## Heat impacts on vulnerable groups

Existing epidemiological studies have shown that the risk of CVD mortality due to heat is predominantly higher in older people (65+), those with underlying health conditions (e.g. hypertension, diabetes, hyperlipidaemia, and coronary artery disease), and socio-economically deprived groups.^85^ A heat vulnerability index has been derived in various countries and is typically computed by factoring in many of these conditions to derive areas that have higher vulnerability to heat stress.^86^ As people age, their ability to maintain thermoregulation decreases, thus increasing CVD risk in hot and humid conditions (42°C, 30–72% humidity). A recent meta-analysis indicates a 0.8% higher RR per 1°C increase in temperature for people aged 65+ compared with those aged 0–64 (1.7% vs. 0.9).^48^ A previous study suggested a 5% higher mortality rate in Germany established for those aged 65–74 and 17% higher for those 75+ compared with those under 65.^87^ A greater risk of heat-related CVD mortality is observed in low-middle-income countries compared with upper-middle-income countries and HICs. These countries often experience high summer temperatures and intense heatwaves, exacerbating vulnerability due to inadequate infrastructure, such as a lack of electricity, air conditioners, and efficient healthcare services during heatwaves.^88^ Recent evidence provides evidence for a 9% higher risk of CVD mortality per 1°C increase in temperature in low- to middle-income countries compared with upper-middle-income countries.^48^ In the multi-country MCC network, higher heat-related mortality from stroke and HF was seen in countries with low GDP per capita. In contrast, higher GDP countries had more heat-related IHD deaths.^45^ Older people with low education levels have a higher CVD mortality risk from heat.^89^ In the USA, climate-vulnerable areas often correspond to historically redlined areas that have often been subject to decades of underinvestment and structural racism.^90^ Additional vulnerable groups include citizens of low-lying island nations of the Pacific and Indian Oceans, indigenous communities in Africa, Australia/New Zealand, and the Americas.^91^

## Heat–drug interactions

Heat can significantly interact with cardiovascular drugs, influencing drug absorption, distribution, elimination, and therapeutic response. Additionally, heat affects the body’s adaptation mechanisms, raising the risk of health side effects. For instance, heat-induced vasodilation can amplify the blood pressure-lowering effects of cardiovascular medications, potentially leading to syncope, serious injuries, or myocardial ischaemia.^41^ Certain medications can impair normal thermoregulatory functions. For example, they can alter cardiac output, sweat rate, peripheral vasodilation, and hydration levels. Anticholinergic drugs inhibit thermoregulation by reducing sweat production, which can cause hyperthermia in extreme temperatures. Diuretics, commonly prescribed for hypertension or HF, can worsen the adverse effects of heat by causing dehydration and electrolyte imbalances. Beta-blockers can also limit the body’s ability to increase heart rate in response to heat, compromising thermoregulation.

Evidence from epidemiological studies highlights the combined impact of heat and medications on hospitalizations and mortality. A matched case–control study of 1405 patients admitted to an emergency department in France during the August 2003 heatwave found that 4% were diagnosed with hyperthermia or heatstroke.^92^ Compared with community controls, those with heat-related conditions had a higher prevalence of anticholinergic drug use, indicating these drugs as an independent risk factor for heat-related hospitalizations. Another study of 345 patients admitted with heatstroke during the same heatwave showed that diuretic use was associated with higher hospital mortality.^93^ These findings underscore the need for heightened awareness among healthcare providers about the risks of heat in patients taking these medications. Preventative measures, such as patient education on staying hydrated, recognizing early symptoms of heat-related illness, and adjusting medication regimens during heatwaves, could help mitigate these risks. Moreover, further research is needed to explore the interactions between heat and other common medications, such as antidepressants and antipsychotics, which may also impair thermoregulation and increase vulnerability to heat-related health issues.

## Heat–health action plans

World Health Organization/Europe recommends countries, regions, and cities to develop and implement HHAP to prevent, manage, and mitigate heat-related health risks.^50^ The HHAPs offer a systematic and comprehensive public health response, encompassing a range of actions at various levels. Heat–health action planning involves collaboration among actors from multiple sectors to manage heat risks better. The guidance of WHO on heat–health action planning is based on eight core elements for effective implementation: agreement on a lead body, accurate and timely alert systems, a heat-related health information plan, reduction of indoor heat exposure, special care for vulnerable population groups, preparedness of the health and social care system, long-term urban planning, and real-time surveillance and evaluation.^50^

These actions should be taken at all levels, from national to institutional (hospitals, care homes, and emergency services). They should be linked to a meteorological early warning system, with specific measures enacted before, during, and in case of a heatwave.

Patients at risk often take medications for underlying CVDs and related risk factors. Commonly prescribed drugs, such as anticholinergics, antihypertensives, antiarrhythmics, antianginals, diuretics, antidepressants, insulin, and certain analgesics, are known to have heat-related risks, including interactions, reduced effectiveness, adverse side effects, and decreased sweating. Medical practitioners must identify at-risk patients, provide behavioural advice, and potentially adjust their medications during high temperatures and heatwaves. Recommendations should also include proper medication storage temperatures (<25°C). Cardiologists are encouraged to offer specific consultations regarding the health effects of climate change, conduct pre-summer medical assessments, and provide advice on hydration and medication adjustments to increase patient awareness and protection.

Urgent research is needed to address prevailing questions, strengthen integrated early warning and disease surveillance systems, implement heat-related health protection measures systematically, and assess their effectiveness. Research should include monitoring health status via wearable devices that can track personalized health measures, including ambulatory blood pressure measurements, arrhythmia, dysglycaemia, and potential interactions with specific medications and comorbidities, particularly Type 2 diabetes and respiratory diseases. Systematic studies on the effects of temperature on medications are critically needed, as empirical evidence is lacking for most drugs.^50^

## Heatwave and specific challenges in cardiac intensive care units

A recent study of American Thoracic Society (ATS) members shows that most ATS members recognize climate change as relevant to patient care and believe that physicians and medical organizations should actively educate patients, the public, and policymakers about its health impacts.^94^ Climate change preparation in the intensive care unit (ICU) will require improved preparedness for extreme scenarios like infectious outbreaks, heatstroke, and other interconnected disasters. There are currently no large initiatives within intensive care medicine to address emerging climate change challenges. However, a range of healthcare organizations are in the process of critically evaluating disaster preparedness, especially related to infectious disease outbreaks, that could serve as a great foundation for climate preparedness, given the overlapping nature of these situations.

## Intensive care unit capacity and special needs during climate disasters

Most ICUs are already stressed on a day-to-day basis with sick patients and shortages of medical professionals and resources, particularly in under-resourced settings. Catastrophic weather events can result in major disruptions to care delivery including interruption of supply chains for equipment and medications, a phenomenon all too familiar during COVID-19 surge.^95^ In one sense, the COVID-19 pandemic may have provided the necessary ‘dress rehearsal’ for health systems to plan for emergencies that could potentially overwhelm the system. The likelihood of unplanned connected cascading disasters owing to climate change is high and may warrant substantial planning. Most hospitals and health systems across the world do not currently have climate mitigation and adaptation plans. Adequate preparation involves increasing ICU bed capacity and enhancing knowledge and infrastructure. Intensive care units need to be ready for sudden surges of critically ill patients during natural disasters, making it essential to monitor ICU bed availability in megacities and coastal areas. Future intensive care staff must be adept at managing uncommon diseases and heatstroke, especially in the elderly. Elevated risks for the elderly were prominent for temperature-induced cerebrovascular, cardiovascular, diabetes, genitourinary, infectious disease, heat-related, and respiratory outcomes during the Europe heatwaves in 2003 and 2023. These risks will likely increase with climate change and global ageing.^6^ Accordingly, the development of local prediction tools, including machine learning/artificial intelligence (AI) approaches, to predict ICU admissions during climate events is very feasible to manage and guide the need to surge capacity. There is a need for comprehensive health impact assessment studies that incorporate several climate variables in addition to social determinants of health, as most climate events will be anticipated to impact the most vulnerable. Intensive care capabilities and services also vary greatly between high- and low-income regions, which could ultimately be a major determinant of a health system’s ability to meet climate change demands and in reducing adverse outcomes.

## Climate change and infectious diseases

Climate change is expected to alter the incidence, prevalence, and distribution of emerging infectious disease (EID) outbreaks, as well as degrade host immunity in humans and human-adjacent species, thereby increasing the potential for more frequent and severe epidemics caused by these pathogens.^19^ The SARS-CoV-2 pandemic and the rapid global circulation of its variant strains underscore the necessity for a collaborative, worldwide framework for infectious disease detection, prevention, and treatment, utilizing newly available technologies. There is an increasing need to forecast the future burden of specific emerging pathogens under various climate change scenarios through models that integrate retrospective data from past epidemics or pandemics and climate conditions.^96^ It is crucial to establish global strategies to track and model potential responses of candidate pathogens to altered climate conditions to predict their future behaviour. This will guide research on early detection, diagnostic technologies, and vaccine development for these targets.

Multi-disciplinary collaborations will be essential to develop effective intercontinental surveillance and modelling platforms employing AI to mitigate the effects of climate change on EID outbreaks through interventions targeting all stages of this process, from the host reservoir onwards. These necessary changes will require coordinated responses and adequate funding support from policymakers, businesses, and healthcare providers to enable effective action from the community level to integrate regional, national, and international efforts.

## Adaptation planning and resilience

**FIGURE 6:**
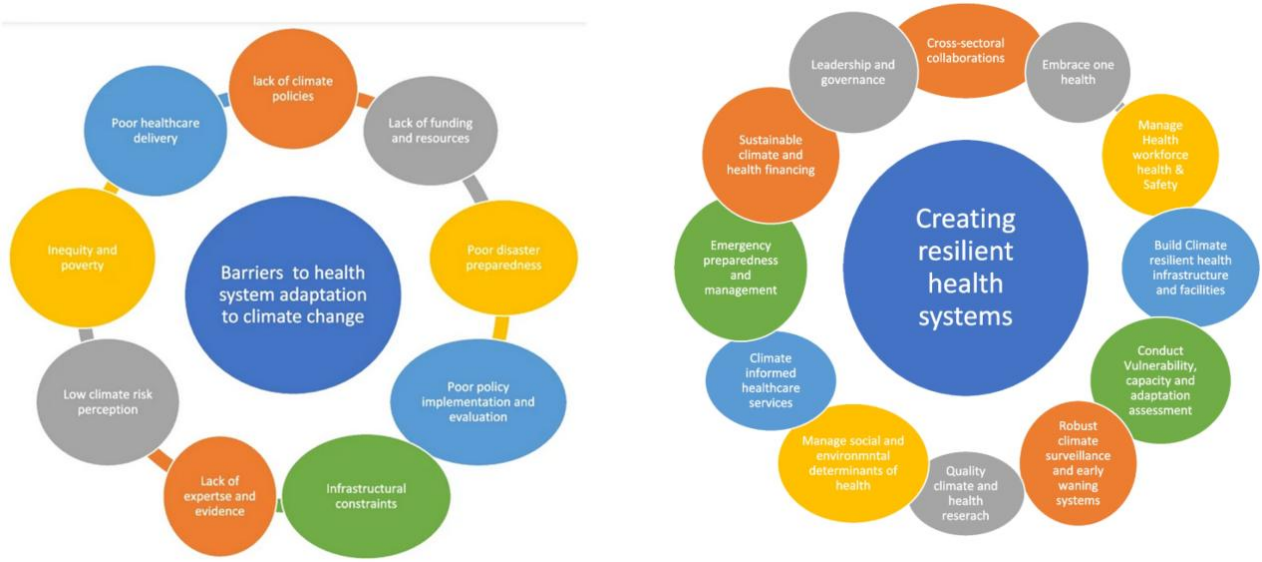
Barriers to health system adaptation to climate change and critical pathways to build resilient health systems. With permission from health systems response to climate change adaptation: a scoping review of global evidence. BMC Public Health volume 24, Article number: 2015 (2024).

The health system plays a central role in climate impact, due to its interconnectedness with public health risks, disease prevention, emergency response, and healthcare delivery to at-risk populations.^19,96^ The WHO Health System Resilience Framework and the new Operational Framework for Climate Resilient and Low Carbon Health Systems, encompass several key components vital to adaptation and fortifying health system resilience to the climate crisis.^97,98^ These include sustainable financing, governance and leadership, health workforce and service delivery, health information systems, medical products and technologies, community engagement, and reduction in carbon emissions. Understanding these elements is crucial for evaluating a health system’s adaptation response and resilience, inducing the ability to adapt and respond effectively to various challenges, including those posed by climate change. Resilient health systems can help absorb and adapt to the challenges posed by climate change, while ensuring essential health services to high-risk or vulnerable populations and promoting sustainable well-being. A recent scoping review found efforts towards building resilient health systems, primarily in developed nations, with investments in technologies and climate-resilient infrastructure, improving awareness and enhancing healthcare delivery and access for high-risk populations, and fostering institutional capacity and robust data systems to navigate the challenges posed by climate change.^99^ But progress is spotty. Across the world, only a minority of countries have initiated plans for adaptation and resilience. *Figure 6* depicts the current barriers to the health system, adaptation to climate change, and critical pathways to build resilient health systems.^99^ Below is a summary of some of the important measures to improve health system resilience and minimize climate-related health hazards.

### Health policy measures

Policy efforts should involve integrating climate considerations into all levels of healthcare policy and planning, incorporating health impact into urban planning, and adding climate change education to school curricula.^100^ Policies for heat risk adaptation, coastal protection, and creating national databases for climate actions, all aimed at ensuring universal healthcare access are needed and are being successfully implemented in many countries in Europe. Effective climate change mitigation and adaptation within health systems depend on long-term planning, collaboration with non-health sectors, sufficient funding, regular policy evaluation, and enforcement of regulations. Unfortunately, most countries, especially in the developing world, at risk for climate-related health impacts lack detailed policy measures.

### Health promotion and communication of climate risks

These measures should include educating communities and health professionals about the health impacts of climate change and environmental pollution and co-benefits of mitigation. The promotion of climate-informed health education programmes improves the community access to these programmes. Effective adaptation also involves emergency risk communication, particularly for high-risk populations, health messaging during extreme events, and rapid, disease-specific emergency responses.

### Disaster preparation, infrastructure, and supply chains

Successful disaster response requires coordination, planning based on reliable information, and sufficient funding. Responses to climate change also include upgrading health infrastructure, ensuring medical equipment resilience, and establishing backup power sources for uninterrupted healthcare during climate events. Health systems are modernizing laboratories for early diagnosis, building social infrastructure like community health centres, and supporting off-the-grid solutions for hospitals. Improving ventilation for thermal comfort and enhancing health supply chains to reduce pollution, quickly identifying needed supplies, and stockpiling essentials are also key strategies. In contrast, developing countries may need to prioritize community-oriented approaches, including public education, clean water access, and building resilience in public health infrastructure.

### Community engagement and integrating primary care

Integrated response plans to address climate-related challenges require input from the community, including the participation of indigenous people, vulnerable communities, and marginalized populations to improve responsiveness, equity, and healthcare quality. Community engagement provides an entry point for health systems to build their social capital, enhance resilience at the individual and community levels, and thereby reduce climate-related health costs. Strengthening primary healthcare and health system governance structures and increasing multi-sectoral coordination and emphasizing local context will enable greater adaptability to address emerging challenges.

### Occupational health and safety

Incorporating climate change effects into Occupational Health and Safety assessments and increasing research on these implications are crucial for adapting health systems to climate change. Ensuring healthcare worker safety involves measures to prevent heat stress, optimize ergonomics, enhance staffing, improve insurance policies, and adjust work processes. Beyond the WHO framework, which focuses on governance and service delivery, additional strategies, including social support systems, and health approaches are much needed.

## Reducing the environmental and carbon footprint of cardiac intensive care units and health systems: a pivot to sustainability in healthcare

A shift in focus from carbon emissions to the broader concept of sustainability is critical. Focusing on carbon emissions alone, without appropriate incentives for behaviour change, is unlikely to accelerate the transition to net-zero emissions.^101^ There continues to be a lack of understanding of the links between carbon emissions and environmental health and appreciation of the concept that what is good for the environment and planet is also suitable for health.^102^ While the health sector is a significant contributor to climate change, it is also an important consumer of resources that contribute to environmental pollution.^103,104^ Consequently, health systems are now striving to lower their carbon footprint and achieve ecological sustainability. The increasing adoption of sustainable practices encompasses three key pillars: economic, environmental, and social practices. It is a much-needed transformation that can accelerate net-zero visions for healthcare.^105^ Placing the economic argument at the forefront of sustainability in healthcare can provide the appropriate incentive to influence the other two pillars. In healthcare, the financial pillar of sustainability, which entails the fostering of alternate practices to reduce costs while maintaining excellent health outcomes, is necessary to ensure uptake and long-term viability. Countries worldwide are experiencing substantial shortfalls in funding for healthcare that does not allow prioritization of resources for climate-related effects unless an economic argument can also be made.^106^

### Reducing the carbon and environmental footprint of intensive care units

The carbon footprint of the ICU is primarily driven by energy use for heating, ventilation, electricity, use of anaesthetics, supplies, and personal protective equipment. Volatile hydrofluorocarbon anaesthetics such as desflurane, sevoflurane, isoflurane, and nitrous oxide (N_2_O) are used during intraoperative cardiac care but not as much in cardiac ICUs and can contribute substantially to warming. The energy intensity of emissions, meaning that the proportion of fossil fuel use for the generation of electricity, can have a substantive impact on emission intensity of similar activities and may account for large regional and national differences in CO_2_ emissions. In a study that estimated ICU emissions, the CO_2_ emissions in both the USA (178 kg CO_2_e/patient vs. 88 kg CO_2_e/patient in Australia) were dominated by energy use for heating, ventilation, and air conditioning (75% of the total), with plastics, nutrition, laundering, ventilator, and other ICU processes and machines contributing only minor emissions.^107^ Identification of areas for resource optimization in the ICU based on local practices and operationalize efficient practices is an important strategy to reduce waste and decease cost.^108^

Interventional procedures are responsible for significant material consumption, all with a carbon and environmental cost.^109^ Most ICUs and interventional procedural areas have pre-made ‘packs’ with items often never used during a procedure. Since many of these items are destined for landfill, there is a substantial opportunity to reduce consumption. Almost all procedures are currently performed without education about carbon footprint or recycling.^110^ A single-centre study in an urban catheterization lab showed that there was a total of 50.6 kg of potentially recyclable material (mostly paper and plastic) collected (mean of 0.72 kg/case), which was highest for percutaneous coronary interventions (1.4 kg/case) and least for right heart catheterization procedures (0.66 kg/case).^111^ Besides the embodied carbon cost of these consumables, there is an associated environmental cost associated with the production and disposal of these products if not appropriately recycled. Customizing ICU procedure kits to reduce waste and redundancy, frugal inventory of devices, and prioritization of equipment utilization including extension and use beyond expiration dates for many devices. Ensuring renewable power is used for both the lab and the washing and sterilizing of equipment, reducing the use of single-use items such as gloves, gowns, and drapes, implementing recycling programmes, installation of energy-efficient equipment and lighting, reducing power utilization during downtimes, and eliminating paper usage.^109^

### System-wide sustainability initiatives

Reducing food waste in hospital cafeterias, reducing fossil fuel use for transportation, reducing travel to meetings, optimizing the use of imaging facilities and shifting to prevention and thereby bypassing carbon-intensive care paradigms are all likely to be very effective, but currently suffer from appropriate incentive models to foster such behaviours.^104,112^ A 2019 Lancet commission report emphasized the urgent need to transform our global food system to achieve sustainable development and climate goals and advocated for a shift towards healthy, plant-based diets and sustainable agricultural practices.^113^ Preventive measures such as lifestyle interventions, and policies to reduce sugar-laden beverages and tobacco sales, which in addition to lowering emissions related to healthcare utilization, can also reduce the emissions associated with the production of these products, and can effectively reduce both environmental pollution and carbon emissions.

## Summary and conclusions

Non-optimal temperature and climate hazards pose disproportionate health risks for CVDs, respiratory diseases, and kidney diseases. Vulnerable populations, especially those with pre-existing CVD, face increased risks of acute cardiovascular events during heatwaves. Age, socio-economic status, and environmental conditions like air pollution further heighten these risks. With global temperatures rising, the frequency and intensity of heatwaves are expected to increase, making it essential to address their health impacts. The body’s thermoregulatory responses to heat stress, such as vasodilation and sweating, can strain the cardiovascular system, especially in individuals with existing health issues. Heatwaves are linked to increased incidences of IHD, strokes, HF, and arrhythmias. Vulnerable groups, including older adults and socio-economically disadvantaged populations, are particularly at risk. Effective public health strategies, such as HHAPs recommended by WHO, are crucial to mitigate these risks. These plans should include early warning systems, education on recognizing heat-related symptoms, and adjusting medications during heatwaves. Intensive care units must be prepared for increased patient loads and the specific challenges posed by extreme heat. Climate adaptation and mitigation plans are going to be critical in reducing morbidity and mortality in response to NOT and extreme events.

## Data Availability

No additional data was included.
